# Collaborative Care for Older Adults with low back pain by family medicine physicians and doctors of chiropractic (COCOA): study protocol for a randomized controlled trial

**DOI:** 10.1186/1745-6215-14-18

**Published:** 2013-01-16

**Authors:** Christine M Goertz, Stacie A Salsbury, Robert D Vining, Cynthia R Long, Andrew A Andresen, Mark E Jones, Kevin J Lyons, Maria A Hondras, Lisa Z Killinger, Fredric D Wolinsky, Robert B Wallace

**Affiliations:** 1 Palmer College of Chiropractic, Palmer Center for Chiropractic Research, 741 Brady St, Davenport, IA, 52803, USA; 2 Genesis Quad Cities Family Medicine Residency Program, 1345 W. Central Park Ave, Davenport, IA, 52804, USA; 3 Thomas Jefferson University Center for Collaborative Research, 720 Edison, 130 S. 9th St, Philadelphia, PA, 19107, USA; 4 Department of Diagnosis and Radiology, Palmer College of Chiropractic, 1000 Brady St, Davenport, IA, 52803, USA; 5 The University of Iowa College of Public Health, S161 CPHB, 101 River St, Iowa City, IA, 52242, USA

**Keywords:** Aged, Chiropractic, Education, Professional, Electronic health records, Family practice, Integrative medicine, Interprofessional relations, Low back pain, Therapy

## Abstract

**Background:**

Low back pain is a prevalent and debilitating condition that affects the health and quality of life of older adults. Older people often consult primary care physicians about back pain, with many also receiving concurrent care from complementary and alternative medicine providers, most commonly doctors of chiropractic. However, a collaborative model of treatment coordination between these two provider groups has yet to be tested. The primary aim of the Collaborative Care for Older Adults Clinical Trial is to develop and evaluate the clinical effectiveness and feasibility of a patient-centered, collaborative care model with family medicine physicians and doctors of chiropractic for the treatment of low back pain in older adults.

**Methods/design:**

This pragmatic, pilot randomized controlled trial will enroll 120 participants, age 65 years or older with subacute or chronic low back pain lasting at least one month, from a community-based sample in the Quad-Cities, Iowa/Illinois, USA. Eligible participants are allocated in a 1:1:1 ratio to receive 12 weeks of medical care, concurrent medical and chiropractic care, or collaborative medical and chiropractic care. Primary outcomes are self-rated back pain and disability. Secondary outcomes include general and functional health status, symptom bothersomeness, expectations for treatment effectiveness and improvement, fear avoidance behaviors, depression, anxiety, satisfaction, medication use and health care utilization. Treatment safety and adverse events also are monitored. Participant-rated outcome measures are collected via self-reported questionnaires and computer-assisted telephone interviews at baseline, and at 4, 8, 12, 24, 36 and 52 weeks post-randomization. Provider-rated expectations for treatment effectiveness and participant improvement also are evaluated. Process outcomes are assessed through qualitative interviews with study participants and research clinicians, chart audits of progress notes and content analysis of clinical trial notes.

**Discussion:**

This pragmatic, pilot randomized controlled trial uses a mixed method approach to evaluate the clinical effectiveness, feasibility, and participant and provider perceptions of collaborative care between medical doctors and doctors of chiropractic in the treatment of older adults with low back pain.

**Trial registration:**

This trial registered in ClinicalTrials.gov on 04 March 2011 with the ID number of NCT01312233.

## Background

Low back pain (LBP) is a recognized public health problem impacting millions of people worldwide. Recent prevalence studies estimate that between one-quarter to one-third of all U.S. adults experience LBP during a three-month time period [1-3]. Studies on the natural history of LBP depict an episodic trajectory of recurrence and recuperation for most people [4], with less than 15% of LBP sufferers either making a complete recovery or experiencing long-term disability at five-years [5]. However, the prevalence of chronic LBP has risen substantially, with an increase in LBP healthcare seeking from 73% in 1992 to 84% in 2006. These increases were documented across all adult age categories [6]. Healthcare expenditures for persons with spine problems also have risen 65% (adjusted for inflation) in the past decade while the impact of spine problems has increased over this same time period [7].

While adults over the age of 65 report LBP prevalence rates similar to persons in the age 45 to 65 age demographic [2,8], the sequelae of back pain in this patient population may have a greater impact on physical function and quality of life than back pain in younger age groups [9,10]. Community-dwelling seniors with LBP have an increased risk of falling, more problems with activities of daily living, such as walking, lifting objects or bathing, greater difficulties in social interaction [9] and are more likely to report depression, poor sleep quality, and more medication use, and to demonstrate decreased performance on functional status tests than pain-free elders [10].

LBP also is among the most common reasons people, including older adults, seek healthcare, either from primary care medical providers [11,12] or complementary and alternative medicine (CAM) practitioners, including doctors of chiropractic (DCs) [13-16]. As such, LBP in older adults presents as an exemplary condition for assessing the feasibility, efficacy, cost-effectiveness, and patient and provider satisfaction with integrative approaches to chronic disease management [17-19]. CAM users note higher levels of satisfaction with their LBP care than do the patients of conventional medical providers [20,21]. Few studies have reported the effectiveness of either primary care treatments [22,23] or chiropractic care [24-28] that include older adults with LBP, much less how these treatments might be combined effectively and safely in this population. The largest controlled trial to date comparing conservative medical management of LBP with two forms of spinal manipulation in 240 patients aged 55 years and older (mean age 63.1 years, SD 6.7) reported higher rates of functional improvement in patients receiving spinal manipulation, with few treatment side effects and no serious adverse effects reported [27].

However, recent studies of care coordination between medical doctors (MD) or doctors of osteopathy (DO) and doctors of chiropractic suggest low patterns of referral or co-management between these provider groups for patients with musculoskeletal conditions [17,29-31]. Among 7,447 Medicare recipients, the percent of episodes of chiropractic-sensitive conditions in which an older adult concurrently visited a DC and another healthcare professional, such as a primary care provider, diagnostic radiologist, orthopedist, anesthesiologist or physical therapist, ranges from 4.9% to 10.9% [32].

Older adults use a variety of self-care, conventional and complementary therapies, including chiropractic care, for pain management, LBP care and treatment of other musculoskeletal conditions [33] as well as for other common symptoms [34]. When concurrent care for older adults with back pain does occur, the patient is often the unit of integration between CAM practitioners and medical physicians, rather than the providers themselves [35]. Problematically, older adult patients may not discuss their use of CAM therapies with their healthcare providers [36], and the use of these therapies may not be documented in medical records [37,38]. When MD/DOs and DCs do make formal referrals to one another, these providers may not exchange important clinical information, such as the patient’s health history, medications, X-ray or laboratory reports, or treatment plans, with one another to facilitate integrative care [30,31,39].

Hsiao and colleagues argue that the appropriate unit of integration for patients seeking care from both CAM practitioners and primary care professionals is at the provider level, not the patient level [35,40]. Four domains for integration between CAM providers and conventional healthcare professionals have been identified: attitudes, knowledge, referral and practice [35]. Similarly, Boon *et al*. present a conceptual framework with seven team-oriented care models ranging from parallel practice to fully integrative, non-hierarchical holistic approaches to practicing patient-centered integrative health care [41,42]. While previous studies have compared LBP outcomes for patients receiving either medical care or chiropractic care [27,43,44], few examples of interdisciplinary medical and chiropractic practice are reported in the literature [45-48], with none evaluating patient-centered outcomes from various clinical practice models of MD/DO and DC collaboration, particularly in the older adult population.

This paper describes the study protocol for the Collaborative Care for Older Adults (COCOA) Study, a prospective, pragmatic, pilot randomized controlled trial designed to compare the clinical effectiveness of three professional practice patterns for the treatment of adults age 65 years and older with subacute or chronic LBP: 1) conventional medical care alone (Med Care); 2) concurrent medical and chiropractic care (Dual Care); and 3) collaborative medical and chiropractic care (Shared Care). Participants in all three groups receive individualized LBP treatments supported by discipline-specific best practice recommendations for the care of older persons with back pain [49-51].

### Primary aim

The primary aim of this pilot study is to evaluate the feasibility and collect preliminary data on the clinical effectiveness of a MD/DO and DC collaborative care model for the treatment of LBP in adults aged 65 years or older. Self-reported back pain and disability are the primary patient-centered outcome measures for this study and the primary endpoint is at 12 weeks [52].

### Secondary aims

The secondary aims are to determine the clinical effectiveness of the three interventions using the following participant-rated outcomes: general health status, fear avoidance behaviors, symptom bothersomeness, depression and anxiety, expectations, improvement, satisfaction, health care utilization, and medication use, with functional status objectively assessed with a brief walking test. Provider-rated expectations for treatment effectiveness and participant improvement in LBP, general health and quality of life will also be measured. As this is a pilot study, the feasibility of the collaborative care model and other clinical trial process outcomes are assessed through qualitative interviews conducted at week 12 with study participants and with research clinicians at the end of their participation in the project. Chart audits of clinician progress notes, and content analysis of clinical trial process notes will also be conducted.

## Methods and design

### Ethics approval

The Institutional Review Boards (IRB) of the Palmer College of Chiropractic (IRB# 2011 G138 – January 19, 2011) and Genesis Health System (IRB# 11–005 – January 28, 2011) reviewed the study protocol and informed consent document and approved the human research participant protection procedures for this trial. The IRBs approve all study protocol amendments and review reportable adverse events throughout the trial. Research clinicians (MDs, DOs and DCs), study coordinators (SCs), the project manager and research nurse, and clinic-based investigators complete the National Institutes of Health (NIH) on-line research ethics training course, *Protecting Human Research Participants,* before interaction with study participants and then on an annual basis, while other investigators complete human subjects training as outlined by their primary appointment institution [53]. Written informed consent is obtained from all study participants.

### Design overview

**Figure 1:**
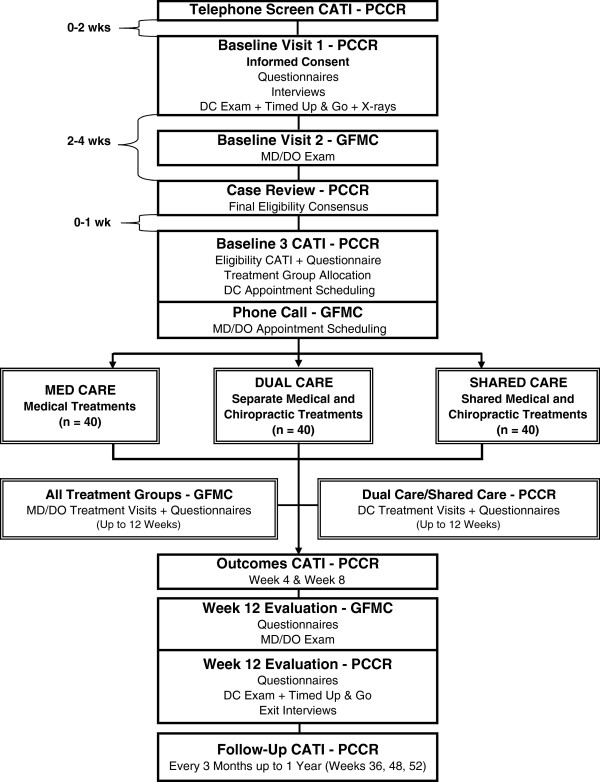
**Participant flow chart.** CATI, Computer-assisted telephone interview. DC, Doctor of Chiropractic. DO, Doctor of Osteopathy. GFMC, Genesis Family Medical Center. MD, Medical Doctor. PCCR, Palmer Center for Chiropractic Research.

The COCOA Study is a prospective, pragmatic, pilot randomized controlled trial [54-56]. Pragmatic clinical trials are designed and ‘aligned with the purpose of comparative effectiveness research’ (p. 208), that is, they enroll patients with diverse background characteristics and common co-morbidities, deliver interventions common in clinical practice, and evaluate patient-centered, clinically-relevant outcome measures [56]. In the COCOA Trial, we are enrolling approximately 120 participants 65 years old or older who report subacute or chronic LBP of a minimum duration of one month. Eligible participants are randomized to one of three parallel treatment groups: 1) conventional medical care provided by a family medicine physician, 2) concurrent medical and chiropractic care provided by an unlinked family medicine physician and a doctor of chiropractic, and 3) collaborative medical and chiropractic care provided by a family medicine physician and a doctor of chiropractic who comprise a patient-centered, co-management team (Figure 1). The collaborative care professionals engage in the following practices to foster interdisciplinary practice: 1) research record sharing via a secure Doctor Communications module specifically constructed and maintained for this study within a web-based Submission, Tracking and Reporting System (STaRS), 2) interprofessional telephone consultations, and 3) patient-centered treatment planning and evaluation. Participants receive up to 12 weeks of individualized, evidence-based medical and/or chiropractic treatments for their back pain [49,50].

Our primary outcome measures are self-reported LBP, measured on an 11-point numerical rating scale, (NRS) [57], and disability measured by the Roland Morris Disability Questionnaire (RMDQ) [58] at week 12. Secondary outcomes include general and functional health status, fear avoidance behaviors, symptom bothersomeness, depression and anxiety, expectations, improvement, satisfaction, health care utilization and medication use. Participant self-report outcomes are measured in person at baseline and 12 weeks, with a subset of outcomes completed at weeks 4 and 8 via computer-assisted telephone interviews (CATI). After the primary endpoint at week 12, we continue to follow participants on selected outcome measures every three months for up to one year (weeks 24, 36 and 52) by CATI. In addition to clinical outcomes, we are describing process outcomes associated with implementing this project across family medicine and chiropractic clinics. We are also assessing participant and provider perceptions of the collaborative care model and the clinical trial design with nested qualitative studies.

### Research settings

This trial is being conducted by investigators and staff at the Palmer Center for Chiropractic Research (PCCR) and Genesis Family Medical Center (GFMC) in Davenport, Iowa, USA. Investigators at The University of Iowa College of Public Health and Thomas Jefferson University Center for Collaborative Research assisted in the design of the clinical study and provide ongoing oversight to the clinical trial through monthly steering committee meetings. Investigators at all four institutions will be involved in data interpretation and the manuscript writing process.

The PCCR was established in 1995 to conduct chiropractic basic science and clinical research. The Office of Data Management and Biostatistics at the PCCR serves as the data coordinating center (DCC) and will conduct the primary data analysis. The Palmer Research Clinic (PRC) of the PCCR is leading participant recruitment efforts, administering the informed consent process, collaborating on baseline assessments, leading the case review process for eligibility determination, contributing to two treatment arms and conducting follow-up assessments.

GFMC is the community-based location of a family medicine residency program accredited by the Accreditation Council for Graduate Medical Education that provides a three-year, post-graduate training program for qualified medical doctors and doctors of osteopathy. The DOs training in this family medicine program are supervised by family medicine medical physicians and do not perform spinal osteopathic manipulative therapy. GFMC is assisting in participant recruitment, collaborating on baseline assessment and eligibility determination, and contributing to all three treatment arms. Both clinics will monitor and address adverse events throughout the study.

### Study population

Participants are recruited from the Quad Cities metropolitan area and select cities in contiguous rural counties of Iowa and Illinois located within 50 miles of the study sites. This region has a combined census count of approximately 568,000 residents, with persons aged 65 years or older comprising 13 to 18% of various county populations [59]. Our target population includes adults aged 65 years and older who currently report subacute or chronic LBP of at least one month duration. We plan to enroll approximately 120 participants for this trial.

### Inclusion criteria

Individuals must be at least 65 years of age with a current episode of LBP lasting at least one month in duration to volunteer for this clinical trial. Participants must demonstrate an ambulatory mobility status by successful completion of the Timed Up and Go Test [60]. Participants also must be willing to enroll in this study regardless of treatment group allocation and provide written informed consent to participate.

In addition to the above inclusion criteria, participants must have a chief complaint of LBP that fits classifications 1 to 9 of the Report of the Quebec Task Force (QTF) on Spinal Disorders (see Table 1) [61]. The treating MDs/DOs and DCs may prescribe or offer LBP treatments within their scope of practice and as recommended by best practice or clinical guideline recommendations. Thus, the QTF classifications in this study are broader than those in other studies of chiropractic care conducted at the PCCR which were designed to either evaluate treatment effectiveness of specific spinal manipulative techniques [27,62] or within a particular subgroup of LBP patients [63].

**Table 1:** **Quebec task force on spinal disorders classification system **[61]

**QTF category**	**Description**
1	Pain without radiation
2	Pain + radiation to extremity, proximally
3	Pain + radiation to extremity, distally
4	Pain + radiation to upper/lower limb with neurologic signs
5	Presumptive compression of a spinal nerve root on a simple roentgenogram (that is, spinal instability or fracture)
6	Compression of a spinal nerve root confirmed by specific imaging techniques (that is, myelography, computerized axial tomography or magnetic resonance imaging)
7	Spinal stenosis
8	Postsurgical status, one to six months after intervention
9	Postsurgical status, more than six months after intervention
10	Chronic pain syndrome
11	Other diagnoses (metastases, visceral disease, recent compression fracture, spondylitis)

### Exclusion criteria

Participants are excluded if they self-report LBP scores of less than 4 on the 11-point NRS at Phone Screen or the Baseline 1 (BL1) Interview or less than 2 at the Baseline 3 (BL3) CATI. LBP diagnosed as Quebec Task Force classifications [61] 10 (chronic pain syndrome) or 11 (other non-musculoskeletal diagnoses) are excluded from this study as these conditions either require additional evaluation or management provided by healthcare professionals outside of the study doctors. Participants who self-report spinal surgery in the past three months or bone fracture in the past six weeks, or who are identified as presenting with a bone or joint pathology (including metastatic bone disease) contraindicating chiropractic care are excluded [64]. Participants are excluded if they self-report any treatment of LBP by a healthcare provider in the past two months or if they are unwilling to avoid LBP treatment from non-study clinicians unless prescribed by their treating clinician during the 12-week trial treatment phase.

Participants also are excluded for the following medical or health status conditions: metastatic disease requiring active treatment; concomitant illness, co-morbidity, mental health conditions, and/or activity of daily living, sensory or mobility impairments that require additional testing, treatment or referral to outside providers, pose safety risks, and/or necessitate coincident medical treatment considered burdensome by the participant; pregnancy; aortic aneurysm of more than five centimeters; daily use of a wheelchair or motorized scooter for locomotion; memory impairment as assessed by the examining clinician during baseline evaluation; or self-reported alcohol or substance dependence or abuse. Participants who are excluded from the study for any clinical concerns are provided with a copy of their baseline evaluation, referral letters (as indicated) and clinical recommendations.

Also exclusionary are current or pending litigation for a work-related injury or personal injury case for LBP; current student, employee or faculty member of Palmer College of Chiropractic or GFMC; current nursing home residence; transportation difficulties; plans to move from the Quad-Cities area during the four months of baseline evaluation and active care; concurrent enrollment in this study by another member of the same household; inability to speak, read or write in English; proxy required to complete any component of the pre-randomization process; or other compliance concerns noted at case review.

### Participant recruitment

Recruitment began in March 2011 with invitation letters sent to current GFMC patients aged 65 years or older who were diagnosed with either of two International Classification of Diseases, 9th Revision (ICD-9) [65] back pain codes (724.2 lumbago and 724.5 backache unspecified) from July 2009 through January 2011. The invitational letter provided an overview of the study and included contact information for calling the PRC if interested in learning more about the study. Invitational letters are also periodically sent to GFMC patients who have recently turned 65 years of age. We also recruit GFMC patients who make visits to their primary care provider for back pain during the study enrollment period. Brochures and flyers are posted at GFMC, PRC and disseminated in campus-wide announcements at the chiropractic college. Additional community-based recruitment strategies include brochure placement in community settings; presentations at community, senior citizen and health-related events and venues; and press releases, articles, and advertisements in local media outlets. Finally, targeted postcard mailers are sent to older adults living within approximately 50 miles of Davenport, Iowa, using a commercial direct mail service [66]. Based upon previous studies conducted by the PCCR in this patient population, we anticipate the inclusion of women and minorities in this clinical trial will be proportional to that in the Quad Cities area [27,66].

### Eligibility determination

It is required that all participants be evaluated by both a doctor of chiropractic and a medically trained physician prior to enrollment as part of our multi-stage eligibility determination process. Interested individuals first contact the PCCR by phone or with the return of a pre-stamped postcard. A staff member administers a short CATI to screen for provisional eligibility and, if the individual is eligible and interested, schedules the volunteer for a BL1 Visit. The study volunteer is mailed a home packet consisting of an introduction letter, appointment reminder with checklist and data collection forms, including demographics, past medical, back pain and chiropractic treatment history, chiropractic beliefs, and health assessments to complete before the BL1 Visit. An appointment reminder phone call is provided the day before the BL1 Visit.

At the BL1 Visit, a SC reviews the informed consent document, flow chart and visit activities with the study volunteer. The study volunteer watches a short video of study procedures, reads the informed consent document and asks questions. Volunteers who do not wish to enroll are thanked for their interest and observe the SC shred the home packet documents in assurance that their protected health information is not used in the study. For participants who sign the written informed consent document, a SC measures the vital signs, height and weight. The participant then completes primary and secondary outcome measurements and a treatment expectations questionnaire. The SC reviews the forms for incomplete data and completes a computer-based form programmed with eligibility criteria. If eligible, the participant completes a medication review, current pain, depression [67] and anxiety [68] questionnaires, and a substance use assessment form to screen for tobacco, alcohol [69] and illicit drug [70] use.

A DC then reviews all health-related research documents, noting areas where further information is needed from the participant or via examination to determine eligibility status or the need for referral. The DC gathers a focused LBP history, conducts an eligibility examination, including a mobility assessment [60] and the World Health Organization Fracture Risk Assessment Tool to evaluate fracture risk [71], and reviews and scores screening questionnaires for depression, anxiety and substance abuse. A dipstick urinalysis (Siemens Multistix® 10 SG, Tannytown, NY, USA) is performed, as indicated. Lumbar spine X-rays may be completed when recent imaging findings are not available to assist in diagnosis, evaluate for the presence of pathology and provide information regarding the safety of available procedures [72]. The lumbar X-rays taken for this study are read and evaluated by the Director of Clinical Radiology at Palmer College of Chiropractic, a DC who has earned a Diplomate from the American Chiropractic Board of Radiology and who is not a member of the research study clinical staff. Additional examinations include auscultation to screen for abdominal aortic aneurysm and assessment of ankle-brachial index for participants with signs or symptoms of peripheral vascular disease. If additional laboratory procedures or diagnostic tests are required to evaluate the participant’s LBP or health status, he or she is excluded from the study and referred to an appropriate healthcare provider for follow-up. With the consent of the participant, health records from outside institutions may be requested and reviewed as part of the eligibility determination.

After the BL1 Visit, the DC completes a web-based eligibility form and writes a brief baseline examination summary which includes demographics, LBP chief complaint, pain profile, health history, X-ray findings, working diagnoses, red flag conditions, and eligibility and clinical concerns for further assessment by the family medicine physician. The BL1 Visit web-based eligibility form includes prompts for the clinician to comment on specific exclusion criteria in a systematic process for each participant as well as an open text field in which to address other possible eligibility concerns. The baseline summary, any outside records and the X-ray report are uploaded to the password protected Doctor Communications web module. These documents and the BL1 Visit eligibility form are reviewed by GFMC physicians before the Baseline 2 Visit.

Participants deemed eligible for further evaluation following the BL1 Visit receive a second eligibility screening exam (Baseline Visit 2-BL2) by a medical or osteopathic physician at the GFMC to assess their general health status, LBP and study eligibility. This exam focuses on age-related concerns such as memory problems, prescription and non-prescription medications, mobility and functional limitations, depression or other mental health concerns, health concerns or findings identified at the BL1 requiring further evaluation by the GFMC doctor, and the LBP chief complaint. The BL2 MD/DO examiner evaluates the participant for other conditions commonly seen in older adults and considered as possible exclusionary criteria, such as cardiovascular disease (abdominal aortic aneurysms, congestive heart failure, and so on) and systemic or organic diseases (infections, cancer, renal or liver impairment, endocrine diseases, and so on). The GFMC physician also completes a web-based eligibility form that includes systematic questions on key eligibility criteria, red flag conditions and open comment fields for additional concerns, and writes the LBP-focused case summary. The GFMC research nurse uploads the case summary document to the password protected Doctor Communications module before Case Review.

Case Review is a twice weekly meeting held at the PCCR that relies upon the combined experience and expertise of DC clinicians, SCs and project managers to: 1) facilitate consistent interpretation and application of the pre-defined eligibility criteria; 2) identify possible red flag conditions, compliance issues, safety concerns, treatment precautions and recommendations for further evaluation or referral; and 3) reach consensus on the participant’s final eligibility status and primary, secondary and co-morbid diagnoses. The research center has used a similar case review process to determine study eligibility of more than 1,100 interested participants in a previous RCT that allocated 221 participants to three treatment groups evaluated the effect of spinal manipulation on sensorimotor functions in back pain patients [62], as well as for clinical trials evaluating medical and chiropractic care for persons aged 55 and older [27,66] and chiropractic care and self-care for back-related leg pain [63].

Briefly, the examining DC clinician provides a verbal presentation and leads a group discussion on the case focusing on the past medical history and clinical presentation, X-ray findings, and the web-based eligibility reports from the BL1 and BL2 visits conducted by the DC and MD/DO respectively, compliance and safety concerns, diagnosis and eligibility determination. An electronic manual of operating procedures is available to reference inclusion and exclusion criteria, operational definitions and study protocols to support consistent eligibility determination decisions, based upon the specific eligibility criteria (for example, aneurysm, carcinoma or metastatic disease, cognitive impairment, alcohol or drug abuse, recent spinal surgery or fracture, QTF classification, safety concerns, and so on) considered for each participant at the Case Review stage of this multi-staged eligibility determination process. Consensus on final eligibility occurs when 80% of the clinicians present agree with the determination decision. If consensus is not reached, the senior clinician will render final eligibility determination. Once eligibility consensus is reached, a Case Review web module programmed with explicit exclusion criteria is completed for each participant and the eligibility decision, along with the reasons for exclusion and comments regarding the Case Review process, is recorded and saved in the Participant Tracking and CONSORT web-modules of the STaRs. An automated e-mail notification is sent to the SCs to contact the participant for treatment group allocation (BL3 CATI). After Case Review, the examining DC calls any participant who is ineligible for study treatments to inform him or her of this determination, review examination findings and offer follow-up recommendations.

### Randomization

Treatment allocation occurs through a 1:1:1 ratio by a predetermined, computer-generated, restricted randomization scheme with random block sizes of three or six allocating participants to one of three treatment groups: a) Med Care, b) Dual Care, or c) Shared Care. Study personnel are blinded to upcoming treatment group allocation. A SC at the PRC accesses the treatment allocation module in the STaRS, selects the participant ID, completes the BL3 CATI to determine the participant’s continued interest in the study and final eligibility for participation, and requests the treatment group allocation. Date and time of the allocation, SC USERID and treatment group allocation are stored automatically in the project database. If the web system is unavailable due to server failure, the backup treatment allocation protocol is administered by predetermined sequentially numbered, opaque envelopes stored in a locked cabinet in a secure location. The participant is told his or her treatment group allocation and then answers a three-item questionnaire about their expectations for back pain improvement, general health status and quality of life. If the participant is allocated to the Dual Care or Shared Care groups, he or she is scheduled for the first DC treatment visit at the PRC. The GFMC Research Nurse receives an automated e-mail notification of the participant’s treatment group allocation and makes a phone call to schedule the first treatment visit at GFMC.

### Study treatments

Participants in all three treatment groups receive up to 12 weeks of standard back pain care from a family medicine physician, while participants assigned to the Dual Care and Shared Care groups also receive up to 12 weeks of chiropractic care. Participants are requested not to seek additional back pain care from non-study providers during the 12 weeks of active care, unless the trial doctors prescribe or recommend such treatments (for example, physical therapy, exercise classes, massage, pain clinic referral). Providers query participants about any outside healthcare appointments, changes in prescribed treatments or self-care activities, and new or resolved symptoms, injuries or adverse effects from study interventions at each treatment visit, with any such changes in health status documented in the paper-based (at PCCR) or electronic (at GFMC) progress notes. Participants are encouraged to continue receiving healthcare for any non-back pain related acute ailments or chronic conditions from their usual primary care providers or medical specialists. In order to limit X-ray exposure among participants, X-ray reports (and images upon request) are exchanged between clinics. The study interventions and availability of other health records are described in further detail below.

### Medical care

All study participants receive conventional medical care from a family medicine resident who is supervised by on-site family medicine physician faculty at the GFMC. Medical care provided in this study includes standard therapies for back pain that occur over a 12-week time period. None of the medical treatments in this study are experimental. GFMC physicians follow the Joint Clinical Practice Guideline from the American College of Physicians and the American Pain Society [51]. The key principles of these recommendations include: a focused history and physical examination; limited diagnostic imaging restricted to select patients (that is, radiculopathy); education about self-management, including maintaining activity levels as tolerated, local heat or cold application, and exercises; pharmacologic management with the use of analgesics and anti-inflammatory agents; and additional therapies that may be applied for participants not responding to the initial interventions, including physical therapy and referral to a pain clinic. All medical care visits are staffed by attending physicians in addition to the resident physicians who are assigned to study participants.

Participants, their insurance company or Medicare are charged for the medical care delivered during the 12 weeks of active care. The minimum dose of conventional medical care for this study is one treatment visit to the family practice doctor. Medical care treatment visits last approximately 15 minutes, with additional visits scheduled as needed. Participants allocated to the Dual Care and Med Care groups receive medical care from a different subset of GFMC physicians than do the participants allocated to collaborative care treatments provided by Shared Care doctors (described below).

### Chiropractic care

Participants assigned to either the Dual Care or Shared Care groups receive chiropractic care at the PCCR Research Clinic for up to 12 weeks. Unlike clinical trials [62,63,73,74] or observational studies [75] of spinal manipulative therapy or other chiropractic techniques, including studies previously conducted with older adult populations [27], clinicians in this study are not limited to any specific type of chiropractic care. This pragmatic design allows clinicians to tailor treatments to the participant’s individual healthcare needs, diagnoses (including QTF classification [61]), priorities and tolerance level, to change treatments over the course of the study as clinically indicated, and for treatments to vary between participants within study groups as they would in clinical practice. When spinal manipulation is considered clinically appropriate, the DC makes decisions regarding the specific technique, application (location and direction), and areas for manipulation [76]. While this study is focused on LBP, participants also may receive chiropractic treatments to other parts of the body, such as the neck, upper back or extremities. The DC may employ one of several forms of spinal or extremity joint manipulation, including but not limited to: 1) high-velocity, low-amplitude maneuvers occurring with direct manual contact, typically resulting in cavitation [77]; 2) low-velocity, variable-amplitude maneuvers occurring slowly and usually repetitively within defined ranges of motion [73,74]; 3) very high-velocity, low-amplitude maneuvers using a mechanical device (Activator® Methods International, Phoenix, AZ, USA) [78]; or 4) passive mobilization [24,79]. All treatment is performed with specialized chiropractic treatment tables (Eurotech, Osage Beach, MO, USA and Thomas Table Company, Davenport, IA, USA). The care provided is comprised of standard therapies commonly used by DCs for the treatment of LBP in older adults; no experimental chiropractic treatments are provided in this study [24,50,80,81]. Chiropractic care also may include rehabilitation exercises (attended or at-home), passive stretching, neuromobilization techniques, manual therapies (for example, ischemic compression, friction massage), counseling (movement, activity, ergonomic and nutrition), recommendations for orthotics, or other modalities (for example, heat, ice, taping, bracing) [82-85]. Rehabilitation exercises may be prescribed using a FitBALL Seating Disc® (Ball Dynamics International, LLC, Longmont, CO, USA) or Thera-Band® equipment (The Hygenic Corporation, Akron, OH, USA) and may include handouts to reinforce teaching (Visual Health Information, Tacoma, WA, USA). As the same group of research clinicians delivers chiropractic care to participants in both the Dual Care and Shared Care Groups, the way this care is delivered should not vary appreciably from a technical standpoint between study participants.

Participants are not charged for the chiropractic care delivered in this study, but may be required to pay on their own or to individually submit bills to Medicare or their insurance companies for co-interventions such as orthotics, monitored exercise programs or special equipment recommended by the DC. The minimum dose of chiropractic care is one treatment visit to the doctor of chiropractic. The typical chiropractic treatment visit is 15 to 30 minutes in duration, with the DC making recommendations regarding treatment frequency (for example, one, two or three times per week) and duration (for example, number of weeks) as the participant progresses through the 12-week active care phase.

### Collaborative care treatments

While both Dual Care and Shared Care participants receive a combination of the medical and chiropractic treatments listed above, only participants in the Shared Care Group are cared for by a doctor dyad composed of a medical or osteopathic family medicine resident physician from GFMC and a doctor of chiropractic from the PCCR who work together along with the participant to determine a shared treatment plan. The Shared Care clinician dyad practices a theory-based, patient-centered, collaborative care model composed of three unique elements: 1) Interprofessional Education, 2) Research Record Sharing and 3) Team Case Management.

Interprofessional Education – The Interprofessional Education Collaborative recently declared interprofessional education and interactive learning among the health professions as essential components for the delivery of safe, high quality, patient-centered healthcare [86]. Core competencies for interprofessional collaborative practice under this initiative include: a) values and ethics; b) knowledge of roles and responsibilities; c) communication; and d) teamwork competencies. These interprofessional practice competencies dovetail with the Hsiao *et al*. model of provider attitudes and knowledge, interdisciplinary referral and integrative practice behaviors that guides this pilot project [35].

An initial group of Shared Care clinicians completed a six-month interprofessional educational program which included cooperative training sessions led by study investigators and other project personnel [87]. Sessions included the following topics: basic educational preparation and advanced training for medical physicians and doctors of chiropractic [88]; LBP overview in older adult populations [50,89]; medical and chiropractic treatments for LBP; and imaging studies for LBP. These didactic presentations were complemented by interdisciplinary discussions of simple and complex cases for LBP suitable for co-management by family medicine physicians and doctors of chiropractic. Additional topics covered included protection of human research participants training [53]; results of focus groups conducted with clinic-based older adults on the topic of LBP and its co-management by family medicine physicians and DCs; and overviews of the clinical trial, including clinical trial fundamentals, manual of operating procedures, STaRS, flow chart, treatment procedures, and data collection forms and procedures.

All Shared Care clinicians also completed a half-day site visit at the partner clinic to job shadow one or more doctors during a typical day treating patients to develop interprofessional collaborative relationships [90]. When time allowed, GFMC doctors joined a case review session for clinical trials currently in progress at the PCCR to observe how BL2 evaluations would support the eligibility determination process. In addition, study investigators led quarterly interprofessional education sessions to discuss clinical trial progress, resolve implementation barriers identified throughout the study, and introduce new collaborative care topics of interest as the study progresses, such as medication management for older adults and rehabilitation options for persons with LBP throughout the trial implementation period. Training boosters on specific trial components and interprofessional communication procedures are provided as needed by the project manager, PCCR senior clinician and GFMC faculty.

Research Record Sharing – The Institute of Medicine has long advocated for the adoption of computer-based patient record systems by healthcare organizations as an essential technology for improving healthcare delivery and patient care outcomes [91]. Key stakeholders in leading integrative medicine centers recommend electronic medical records as a critical administrative structure for successful integration of CAM and conventional therapies in healthcare organizations [92]. Previous studies also have identified a need for shared clinical documentation, including case reports, X-rays and other imaging studies, and additional clinical information, to improve continuity of care, treatment coordination and interprofessional referrals between primary care doctors and chiropractors [30,31]. Thus, an innovative infrastructure component of this collaborative care model is the use of a web application Doctor Communications module to allow secure, convenient availability and access of participant research records between Shared Care clinicians located at the two geographically distant and institutionally distinct research sites. Shared Care clinicians may access the following research records throughout a participant’s involvement in the active care phase of the trial: past medical and chiropractic history, pain history, medications checklist, radiology reports (if available), outside medical reports (when present), BL1 and BL2 exam summaries, treatment summaries, telephone consultations and change of status reports. Shared Care clinicians and support personnel receive automated e-mail notifications when research-related documents are uploaded and available for review.

Team-Based Case Management – Each Shared Care participant is assigned to a team of treating clinicians (MD/DO and DC) who follow the participant’s progress throughout the 12-week active care phase. Some participants may receive care from more than one MD/DO or DC due to scheduling logistics. Following the participant’s first treatment visits to both the GFMC and PCCR clinics, the Shared Care clinicians each prepare and upload initial case summaries onto the secure website, and arrange an interprofessional telephone consultation to discuss the participant's past health history and current case presentation, individual goals, anticipated progress and treatment recommendations. A Shared Care treatment plan is mutually established and the doctor who next sees the participant discusses these recommendations with the participant and incorporates any feedback into the overall treatment plan and communicates such changes to the Shared Care treating partner. Each Shared Care clinician is familiar with the treatment plan and provides the participant with encouragement, advice and support for both the individual’s goals and the general direction of the co-management treatment plan [93]. Additional telephone call consultations or research record exchanges may be initiated for Shared Care participants by either treating clinician throughout the study as the participant's condition, response to medical or chiropractic treatment, or need for referral to primary care provider or health specialists warrant.

### Outcome measures

Outcomes are measured by participant self-report, objective assessment, and in-person and telephone interviews. Outcomes are collected at baseline, 4, 8 and 12 weeks (primary endpoint), and every 12 weeks thereafter for up to one year (weeks 24, 36 and 52). The baseline visits and 12-week evaluations are scheduled as in-person visits at each clinic. At these visits, a SC administers the self-report questionnaires and interviews to the participant and cleans the data per protocol. Study clinicians (MD/DO and DC) perform the objective clinical assessments. DC exams are conducted by examining clinicians who do not treat participants as part of this trial; GFMC treating physicians complete the 12-week MD/DO exams with their assigned participant to maintain continuity of care. Due to logistical constraints with the doctors’ schedules, pre- and post-evaluations may not necessarily be completed by the same clinicians. A semi-structured qualitative interview is conducted by the project manager or SC at the PRC during the 12-week evaluation after completion of all other assessments to understand the participant’s perceptions of back pain improvement, clinical trial experience, expectations, treatment effects and interprofessional collaboration among the treating clinicians. We attempt to obtain outcome data from all trial participants, even those who decide to not attend treatment visits, drop out of care or move away from the area. If a participant is unable to attend the 12-week visit in person, the primary outcome measures and a subset of secondary outcomes are administered via CATI or paper-based questionnaires sent to the participant’s home with a stamped return envelope.

### Self-report outcome measures

Participant-rated pain is a primary outcome measure [52]. Participants rate their average level of LBP in the past week and their worst level of LBP in the past week on an ordinal, 11-point numerical rating scale (0 = no LBP; 10 = worst LBP possible) during the phone screen; at the BL1 and BL2 Visits, and BL3 CATI; and all follow-up assessments [94]. The NRS has excellent metric properties, is easy to administer and score, and is used often in LBP research [95,96]. The minimal clinically important difference (MCID) for the pain NRS is a change of 2.5 points [96].

Participant-rated LBP disability also is a primary outcome measure. We use the 24-item Roland Morris Disability Questionnaire to assess LBP-related disability [58,97,98]. The one-page RMDQ is among the most common and respected LBP assessment instruments in LBP outcomes research and exhibits well-documented reliability and validity [52]. The RMDQ can discriminate between different forms of treatment for back pain and is sensitive to clinical change [99,100]. The MCID of the RMQD is estimated at two points [101]. We are piloting three LBP-disability questions not captured by the RMDQ but that represent common functional limitations in LBP populations: difficulty completing moderate exercise, normal work activities and driving a motor vehicle [102].

Participant-rated secondary outcome measures include general health status, fear avoidance, symptom bothersomeness, depression and anxiety, functional mobility, expectations, improvement, satisfaction, health care utilization and medication use.

General health status is collected with the Veterans RAND 36-item Short Form Health Survey [103,104] to assess changes in physical functional and mental health from baseline levels at 4, 8 and 12 weeks of care.

The Fear Avoidance Beliefs Questionnaire (FABQ) is a 16-item instrument measuring back-pain fears on two subscales related to Physical Activity and Work which are rated from 0 (completely disagree) to 6 (completely agree). The FABQ has demonstrated reliability and validity in chronic LBP populations, with lower FABQ scores indicating the probability of successful treatment with spinal manipulation [105-107]. The FABQ is used to assess change in LBP fears from the BL1 visit to the 12-week evaluation.

Symptom bothersomeness is a common quality of life measure for LBP studies. Participants rate the bothersomeness of their back pain symptoms as well as any depression symptoms during the past week, with each symptom measured on a 1 to 5 scale, where 1 = not at all bothersome and 5 = extremely bothersome. A symptom bothersomeness index is calculated by summing five symptom ratings. Bothersomeness questions are practical and have demonstrated internal consistency, construct validity and responsiveness to change in patients with LBP [108].

Mental health outcomes are measured at baseline and week 12 with the Patient Health Questionnaire-9 for depression and the General Anxiety Disorder-7 for anxiety [67,68]. The Patient Health Questionnaire-9 is a brief screening instrument based upon the Diagnostic and Statistical Manual (DSM-IV) and adapted from studies for assessing depression symptoms and severity in primary care settings [109]. The nine-item tool is scored on a 0 to 3 scale with total scores ranging from 0 to 27 and a MCID of five points. The General Anxiety Disorder-7 is a short, self-report instrument for the clinical assessment of generalized anxiety disorders [68]. The seven-item tool is scored on a 0 to 3 scale with total scores ranging from 0 to 21.

Functional mobility is measured at baseline and week 12 with the Timed Up and Go Test [60]. This brief screening tool is used to identify older adults with poorer functional mobility and those who are prone to falling [110]. During the test, the participant rises from a chair, walks 10 feet, turns around, walks back to the chair and sits down while the observer times the trial.

Health care utilization is based upon participant self-reported estimate of their use of additional health care services and out-of-pocket costs for LBP, including the provider types, number of visits, and description of services or products at baseline and all follow-up assessments.

Medication use, including current non-prescription and prescription medication as well as dietary and nutritional supplements, is assessed by participant self-report at baseline and all follow-up time points.

Participant satisfaction with care received during the trial is rated on an investigator-designed instrument that is based on common elements of satisfaction reported in the LBP literature [111]. Overall satisfaction with care is rated on an 11-point NRS with the anchors “not at all satisfied” to “extremely satisfied”. Participants also rate their satisfaction with seven different aspects of back pain care on a five-point Likert scale (1 = poor; 5 = excellent): information received regarding the cause and prognosis of LBP; information received regarding activities that would hasten recovery and prevention of future LBP problems; concern shown by the provider; quality of treatment recommendations; and overall care for LBP. Participants also complete a seven-point Likert scale (0 = very poor; 6 = the best) that assesses their satisfaction with clinic convenience, as well as the technical skills, listening skills, and the amount of time spent with the clinicians and research staff. For participants in the Dual Care and Shared Care groups, care satisfaction is rated for both the treating DC and MD/DO, while Med Care group participants only rate their satisfaction with medical care.

Participant expectations of treatment and LBP improvement are assessed with an adapted questionnaire from a study of the effect of expectations on the evaluation of effectiveness of acupuncture and massage among LBP patients [112]. Participants rate the perceived helpfulness of five treatment options (for example, medical care, chiropractic care, physical therapy, concurrent medical and chiropractic care, and collaborative medical and chiropractic care) for improvement of their LBP on an 11-point NRS with the anchors “not at all helpful” to “extremely helpful” at the BL1 Visit. Participants also rate their expectations about LBP improvement three months from baseline on a seven-point scale anchored with “completely gone” to “much worse”, as well as expectations for improvement in their overall health status and quality of life on a seven-point scale anchored with “very much improved” to “very much worse”. We repeat the questions about expected improvements in LBP, overall health status and quality of life during the BL3 CATI to assess whether participant expectations for improvement change after allocation to a treatment group. At the 12-week visit, participants rate the level of improvement they achieved in each of these three domains since the start of the study.

Clinician expectations of care and LBP improvement: Because this is among the first studies to evaluate the benefits of collaborative care between medical doctors and doctors of chiropractic in the treatment of patients with chronic LBP, we also are interested in clinicians’ expectations of care and LBP improvement for these participants [113]. The DC and MD/DO clinicians who perform the baseline examinations rate expected improvement from each of the five LBP treatment modalities on the same 11-point NRS described above for participant expectations. These clinicians also rate their expectations for improvement in the participant’s LBP, overall health status, and quality of life on the same seven-point scales identified above. Examining clinicians complete a brief narrative text box to describe their rationale for the care and LBP improvement expectations they hold for the participant. Treating DC and MD/DO clinicians complete these same questionnaires after the first treatment visit.

### Blinding

As this is a RCT evaluation for three models of interprofessional practice between medical physicians and DCs, the study participants, study coordinators or treating clinicians are not blinded to the participant’s treatment allocation. The primary and most of the secondary outcome measures are self-administered questionnaires distributed by the study coordinators. Participants complete these questionnaires on their own in a consult room at the Baseline Visit 1 and at the Month 3 evaluation. The Timed Up and Go Test is completed at these visits by an examining clinician who has not provided care to the participant during the 12-week active care phase of the study. Other follow-up assessments are collected via CATI conducted by trained interviewers who do not provide study treatments and who are masked to participant outcomes assessment data from all other time points. Study clinicians remain blinded to participants’ outcome data for the entire study period. All analysts and study investigators are blinded to treatment group until data analysis is completed.

### Qualitative interviews

Participants complete an individual qualitative interview at the end of the 12-week active care phase following all other outcome measures [114]. A semi-structured interview schedule guides the interview process with follow-up probes used to elicit the participant’s unique experiences in the clinical trial. In most cases, the project manager (who does not have day-to-day contact with participants) completes the interview session to encourage participants to speak freely about their experience in the study. When the project manager is not available, a SC trained in the interview protocol conducts the interview. Participants are asked questions about their interest in the clinical trial; overall experience in the study; treatment expectations and effect of treatment allocation on those expectations; perceived changes in back pain and other health conditions during active care; potential impact of illnesses, injuries or other setbacks on LBP progress and quality of life; treatment likes and dislikes; relationship with medical doctor and/or doctor of chiropractic; and perceptions of the collaborative care process. Participants provide written consent at the time of the informed consent procedure and verbal permission to audio-record the qualitative interview at the start of the session. To assure confidentiality, interviews are conducted in a private consult room with the door closed, participants are not identified by name during the interview, and only the participant ID number marks the audio-recording and transcripts. In addition, we conduct exit interviews with interested medical physicians and DCs who provided care to study participants to determine their perceptions of the feasibility and sustainability of the study model. All interviews are transcribed verbatim by an independent transcription service, reviewed for completeness and accuracy by the project manager, and imported into a qualitative data analysis computer database (NVivo-9, QSR International Pty Ltd., Doncaster, VIC, Australia) for analysis.

### Data management

The DCC handles the data management procedures for this clinical trial using modifications of data handling procedures described elsewhere [62]. The STaRS is comprised of multiple sub-modules integrated into one comprehensive study management web application that includes CATIs for phone screens and follow-up data collection, participant eligibility checks, treatment allocation, participant tracking, a tickler system, report generation and scheduling. The ASP.NET web application elements were programmed in C# and Structured Query Language (SQL) using Microsoft Visual Studio 2005 (Microsoft, Redmond, WA, USA) and Microsoft SQL Server Management Studio (Microsoft). User-friendly data entry interfaces were programmed with appropriate participant flow restrictions, validation schemes and skip patterns. The STaRS interfaces with the Central Participant Database and uses a Project/Users Permissions System to control project personnel access to web modules. The DCC added the Doctors’ Communication module to facilitate the secure transfer of participant research records and to provide file posting updates with automated e-mail notification to the DC and MD/DO clinicians who collaborate on LBP care for participants in the Shared Care group, as well as to the GFMC research nurse and PCCR project manager and senior clinician.

Participant self-report questionnaires and clinician-completed progress notes are paper-based forms. Research outcomes data collection paper forms include only the unique numerical participant ID as an identifier. Study coordinators provide oversight of all paper data collection forms, log each completed form into the form tracking module of STaRS, and submit data forms for key-entry. The project manager transfers paper records from the medical clinic to the DCC on a weekly basis. Forms are also transferred between clinics on secure fax systems. Paper data collection forms and participant research record charts are stored in locked filing cabinets at each clinic. Trained data-entry clerks double key-enter paper forms in a Microsoft Windows® (Microsoft) program using range and validation checks.

The STaRS web system is password-protected and uses a Microsoft® SQL Server (Microsoft) database platform to store all data. Web programmers collaborated with project personnel to gather system requirements and designed the web applications and database structures based on PCCR standards. DCC personnel developed the electronic data collection forms, programmed web applications to support data and project management, provide data form key-entry and technical support, and execute procedures for data security and data quality control, storage and back-up. The Data Manager maintains the data dictionaries and creates the datasets for analysis.

Participant enrollment data are collected at each stage of the clinical trial from initial phone screen through treatment allocation to the 12-week primary endpoint and 52-week follow-up period, so participant flow is reported according to CONSORT guidelines [62]. Specifically, we collect recruitment source, total responses per recruitment source, race/ethnicity and gender distribution, disposition status (for example, ineligible, does not wish to participate, allocated, withdrawal, completed active care, completed follow-up, lost to follow-up), reasons for non-participation or exclusion (for example, current treatment, cost, treatment allocation, perceived benefit, travel, health concerns, baseline exam results, multiple/other and so on), and the number allocated to each treatment group [66].

Data management and quality control of both key-entered forms and web forms are performed using SQL views, stored procedures and real-time, web-based reports. Automated reports are viewed by the data manager and project manager to determine if quality improvement actions must occur, such as improved documentation, protocol revisions or personnel retraining. Final project datasets of combined web and paper data are assembled by transferring data from Microsoft® SQL Server (Microsoft) to SAS System for Windows (Release 9.2, SAS Institute Inc., Cary, NC, USA). The data manager writes and tests SAS programs to create datasets as requested by the investigators and creates the data dictionaries. DBMS Copy (Conceptual Software, Houston, TX, USA) software is used to move the data across software applications.

### Data and safety monitoring

A Data and Safety Monitoring Committee (DSMC) provides scientific and ethical oversight on all ongoing human clinical trials conducted by the PCCR. The DSMC currently is composed of epidemiologists with expertise in LBP and/or CAM clinical trials, a medical physician, a doctor of chiropractic and a biostatistician, none of whom are employed by PCCR or GFMC. The DSMC approved the study protocol, informed consent document and human research participant protection procedures before trial implementation. To monitor trial data, the DSMC reviews status reports prepared by the trial biostatistician that include recruitment accrual, enrolled participant baseline characteristics and other enrollment data; data collection form processing; follow-up assessment completion; treatment compliance; study protocol deviations and other clinical trial data. To monitor participant safety, the DSMC reviews reportable adverse events (see below) every six months and assesses any serious adverse events upon report receipt. No formal stopping rules were established in advance of trial implementation. The DSMC may make recommendations to the principal investigator and funding agency regarding study progress, termination or trial modifications. We also report serious adverse events to the funding agency following receipt of the report.

### Adverse events and UPIRTSOs

An IRB-approved adverse event (AE) grading and reporting protocol defines the processes by which AEs are monitored and categorized in this clinical trial. This protocol also outlines when and how participant safety concerns are reported to the IRBs, DSMC and funding agency. An AE is defined as any untoward medical occurrence that may present itself during the conduct of the study and that may or may not have a causal relationship with study procedures [115]. A serious adverse event is defined as an adverse event resulting in any of these outcomes: death, a life-threatening adverse experience, hospitalization or significant disability/incapacity [115]. A previous clinical trial of six weeks of chiropractic care or medical care for persons over age 55 years with LBP reported no serious adverse events associated with any of the interventions and only 21 side effects in 20 participants among 240 randomized participants [27].

Our research center uses an active process of clinical surveillance [116] to monitor adverse events and other changes in participant health status throughout the course of our studies. Research clinicians, SCs and the GFMC research nurse receive ongoing training on AE identification, grading, reporting and monitoring. Participants also are instructed to report new symptoms or changes in health status to the research team at each treatment visit and to contact the clinics in the event of pain, discomfort or other clinical concerns they believe may be related to study treatments. Research clinicians monitor participant safety by conducting clinical evaluations of health status and querying participants for new symptoms or other changes in health status, alterations in prescribed or self-care treatments, and the development of any untoward effects from study interventions at each treatment visit and all follow-up assessments.

When AEs are suspected, the treating DC at the PRC or the research nurse at GFMC completes an AE monitoring form which includes a description of the event, date of onset, impact of prognosis and referral to outside care. The clinician or nurse then rates whether an AE is: 1) mild, moderate, severe or serious in severity; 2) expected (disclosed in the informed consent document or part of an underlying disease) or unexpected (more serious than expected, or not disclosed in informed consent document); and 3) related to the intervention (for example, definitely, probably, possibly, unlikely or unrelated). AEs are followed to resolution at which time an AE resolution form is completed. An automated web-based system records all AEs to allow the project manager and senior clinician to track and report adverse events to the principal investigator, both IRBs of record, DSMC and funding agency.

We also monitor and report Unanticipated Problems Involving Risks to Subjects or Others (UPIRTSOs). Federal regulations (45CFR46.103(b)(5) and 21CFR56.108(b)(1)) require study investigators to promptly report UPIRTSOs to local IRBs. The Palmer College of Chiropractic IRB defines UPIRTSO as any problem or event that in the opinion of the local investigator was unanticipated, serious and at least possibly related to the research procedures.

### Sample size justification

The sample size of this pilot randomized controlled trial was selected as a minimum of 120 participants with equal allocation to each of three treatment groups. This sample size should provide adequate participant contact in each treatment group to assess and refine the collaborative care model, determine the feasibility of conducting a full-scale trial and obtain reasonable estimates of effect sizes and variability to use in powering a full-scale trial. Although the sample size was not chosen to detect between group differences, the power of the study to detect differences of 2 points on the RMDQ and 2.5 points on the NRS with 40 participants per group, assuming at least 85% of participants complete the 12-week primary endpoint assessment, will exceed 75%.

### Data analysis

DCC biostatisticians will conduct the quantitative data analyses using SAS System for Windows (Release 9.2) (SAS Institute Inc.) and S-Plus 8.0 for Windows (TIBCO Spotfire, Somerville, MA, USA). Co-investigators with expertise in interpretive methodologies will conduct qualitative data analyses of the individual interviews with study participants using NVivo-9 qualitative data analysis computer software (QSR International Pty Ltd., Doncaster, VIC, Australia). Both analytic teams will collaborate with the investigators in presenting and interpreting the results.

### Quantitative data analysis

Descriptive statistics of participant baseline characteristics will be presented for each treatment group to assess their comparability. Descriptive statistics of the primary and secondary outcome variables will be presented for each treatment group at baseline, and weeks 4, 8 and 12 (primary end point), and for follow-up measures at weeks 24, 36 and 52.

We will estimate mean effects of the primary outcome variables by modeling over weeks 4, 8 and 12, adjusting for the baseline measure. Linear mixed models will be used to examine patterns and estimate effects between groups after fitting models that account for the correlation across measurements in the same participant. The adjusted mean differences and 95% confidence intervals between groups at week 12 will be based on the final models. Intention-to-treat analyses will be used; the analyses will use data for every time point that a participant completes.

Point estimates with 95% confidence intervals will be reported for pairwise differences between groups on the secondary outcome variables. We will not control for multiple comparisons in this study, but will use these pilot data to determine the most efficient design and estimate the sample size needed, accounting for multiplicity issues, for a full-scale trial. Although we will not impute missing data for analysis of these data, we will examine for patterns of missingness to guide the missing data analysis plan for a full-scale trial.

### Qualitative data analysis

We will analyze individual interview transcripts through a two-stage process using conventional and directed content analysis techniques [114]. First, we will use an inductive approach to identify broad topics in the transcribed text, coding each question to identify such themes as motivations for study participation; treatment expectations; collaborative care process; doctor-patient relationship; treatment adverse effects; and perceived treatment benefits. For topics with more substantive theory, we will use a template-style coding process to create a code manual, computer code the text, sort similar text into groupings for reading, and verify coding structure [114]. We will compare participant responses within and across treatment groups to identify areas of consensus and variation in participant experience. Credibility of qualitative coding will be established through prolonged engagement with the texts and coding process, triangulation, and the use of multiple coders and peer debriefing [117].

## Discussion

This pilot study protocol describes the design of an innovative randomized controlled trial to evaluate the clinical effectiveness and feasibility of three professional practice models for the treatment of older adults with low back pain by family medicine physicians and doctors of chiropractic. To the best of our knowledge, this randomized controlled trial is the first to compare patient-centered outcomes for older adults with LBP receiving conventional medical care, co-occurring medical and chiropractic care, or collaborative medical and chiropractic care. Our study design has several strengths. The study protocol is based upon interdisciplinary evidence-based, clinical practice guidelines for the care of patients with back pain. The population under study, older adults aged 65 years or older represent some of the most challenging LBP patients as they often present to their primary care providers and doctors of chiropractic with multiple co-morbid conditions and decades-long treatment histories. Our rigorous eligibility determination process paired with individualized medical and chiropractic treatments allowed the inclusion of complex back pain patients as participants in this study, which reinforces its pragmatic design. In addition, our multi-stage eligibility process is designed, described and documented so that an independent group of researchers, if they followed the description provided, would recruit sufficiently similar participants. With further development and refinement, the exploratory model of provider-level integrative medicine used to guide the interprofessional collaboration between the family medicine physicians and doctors of chiropractic in this study may serve as an example for healthcare organizations seeking to integrate conventional and CAM services in real world settings.

## Trial status

Participant recruitment began 04 March 2011. The first participant was allocated on 05 April 2011. The final participant was allocated on 21 August 2012. The active care phase of the trial ended in November 2012. The six-month follow-up period will end March 2013.

## Abbreviations

AE: Adverse event; BL1: Baseline 1 visit - PCCR; BL2: Baseline 2 visit - GFMC; BL3: Baseline 3 - treatment allocation CATI; CAM: Complementary and alternative medicine; CATI: Computer-assisted telephone interview; COCOA: Collaborative Care for Older Adults; DC: Doctor of chiropractic; DCC: Data coordinating center; DO: Doctor of osteopathy; DSMC: Data and Safety Monitoring Committee; DSM-IV: Diagnostic and Statistical Manual; Dual Care: Concurrent medical and chiropractic care group; FABQ: Fear Avoidance Beliefs Questionnaire; GFMC: Genesis Family Medical Center; ICD-9: International Classification of Diseases 9th revision; IRB: Institutional review board; LBP: Low back pain; MCID: Minimal clinically important difference; MD: Doctor of medicine or medical doctor; Med Care: Conventional medical care group; NIH: National Institutes of Health; NRS: Numerical rating scale; PCCR: Palmer Center for Chiropractic Research; PRC: Palmer Research Clinic; QTF: Quebec Task Force; RMDQ: Roland Morris Disability Questionnaire; SC: Study coordinator; Shared Care: Collaborative medical and chiropractic care group; SQL: Structured Query Language; STaRS: Submission tracking and reporting web-based system; UPIRTSO: Unanticipated problems involving risks to subjects or others.

## Competing interests

The authors declare that we have no competing interests.

## Authors’ contributions

CMG, SAS, RDV, CRL, AAA, MEJ, KJL, MAH and RBW participated in the conception of the trial. CMG, SAS, RDV, CRL, AAA, MEJ, KJL, MAH, LZK, FDW and RBW participated in the design of the trial. CMG, SAS, CRL and RBW participated in plans for the analysis of the data. CMG, SAS, RDV, CRL, MEJ, MAH and RBW drafted this manuscript. CMG, SAS, RDV, CRL, AAA, MEJ, KJL, MAH, LZK, FDW and RBW provided critical review of this manuscript. All authors read and approved the final manuscript.
